# Prevalence of Diabetes Mellitus and its Related Factors in Patients with Tuberculosis

**DOI:** 10.34172/aim.2022.129

**Published:** 2022-12-01

**Authors:** Hossein Tireh, Hamid Heidarian Miri, Nasim Khajavian, Amin Samiei, Monavar Afzalaghaee

**Affiliations:** ^1^Student Research Committee, Mashhad University of Medical Science, Mashhad, Iran; ^2^Management & Social Determinants of Health Research Center, Mashhad University of Medical Sciences, Mashhad, Iran; ^3^Instructor Social Determinants of Health Research Center, Gonabad University of Medical Science, Gonabad, Iran; ^4^Tuberculosis Coordinator at Health Department of Mashhad University of Medical Sciences, Mashhad, Iran

**Keywords:** Diabetes mellitus, Iran, Prevalence, Tuberculosis

## Abstract

**Background:**

Considering the high prevalence of tuberculosis (TB) in developing countries and the fact that comorbidity with diabetes mellitus (DM) imposes a higher burden on the society, this study was carried out to assess the prevalence of diabetes and its related factors in patients with TB in Mashhad, Iran.

**Methods:**

In this study, we enrolled 405 patients over the age of 18 who had been diagnosed with TB between the years 2015 and 2016 according to the documents of the ministry of health. The participants were selected randomly from five health center domains based on the stratified sampling method. The patients were screened for diabetes according to HbA1c over 6.5% or a fasting blood sugar (FBS) level over 126 mg/dL at different time points and the patient’s self-report of having DM.

**Results:**

The mean age of participants was 51.14±20.52 (19–92 years). The prevalence of DM in patients with TB was 21.2%, of whom 3.5% were newly diagnosed. Among potential factors, age with OR=3.786 (1.183, 12.113), body mass index with OR=9.149 (3.182, 26.302), nationality with OR=2.149 (1.122, 4.117) and TB type with 3.328 (1.44, 7.689) were associated with DM in patients with TB.

**Conclusion:**

The prevalence of DM in our study was higher than that observed in other countries. Our study showed associated factors like age, body mass index, and TB type to be very important. Also, the prevalence of DM was different in patients with different nationality.

## Introduction

 Diabetes mellitus (DM) and tuberculosis (TB) are among the oldest known diseases in humans. which still have high incidence and prevalence despite the many advances made regarding their prevention and treatment.^1^ TB is the most common cause of death due to infectious microbial diseases worldwide,^2^ while diabetes is known as a deadly disease which kills around 6 people every minute.^3^

 Several studies have shown that the prevalence of DM and TB in developing countries with moderate or low income is higher than other countries,^4^ as 95% of TB patients and over 70% of DM cases live in such nations.^5^ The association between DM and TB was recognized in the early 20^th^ century.^6^ Since then, studies performed all over the world have shown DM comorbidity in 12%–44% of TB cases, whereas the prevalence of diabetes in TB are around 1.9% to 35%. Today, the concurrence of these two diseases is regarded as a threat for the global health.^4,5^

 TB and diabetes in Iran, as a developing nation, have a considerable prevalence; this figure is 8%–15% (22 in 100 000 individuals in Razavi Khorasan) of TB and up to 24% for diabetes.^2,7,8^

 In a study conducted in Tehran, Iran in 2012, the prevalence of diabetes in TB cases was 34.5%.^9^ Regarding other countries, the prevalence of diabetes in those infected with TB was 8.3% in Ethiopia, 6.3% in China, 25.3% in India and 44% in Kerala city, India.^4,5,10,11^

 It seems that the association between DM and TB is the next challenge for the global control of TB. Therefore, understanding the two-way relationship between these two diseases is necessary for proper planning and global collaboration on reducing the dual burden of DM and TB.

 This study aimed at investigating the prevalence of diabetes in patients with TB in a 2-year period in the city of Mashhad, so that by understanding the relationship between these two diseases, a controlling program can be proposed for patients with TB in order to better manage the risk of diabetes and its complications.

## Materials and Methods

 In this study, we enrolled 405 patients over the age of 18 who had been diagnosed with TB between the years 2015 and 2016, based on the global directly observed therapy (DOT) guideline.

 Mashhad city health centers were divided into five main domains based on the Deputy of Health classification. The participants were selected from these five domains based on the stratified sampling method. In other words, each health center was regarded as a class and a few records were randomly selected, according to the population affected by TB, in each center of these classes. Some of the baseline data of the patients were collected by a questionnaire consisting of the demographic data, lifestyle, clinical data, type of TB and their treatment status. The patients were then screened for diabetes according to the WHO guidelines.

 Based on these criteria, and due to the comorbidity of TB and DM, for all TB patients, DM testing and routine evaluation are required; patients with HbA1c over 6.5% or a fasting blood sugar (FBS) level over 126 mg/dL at different time points were selected as the target group. The patient’s self-report of having DM was considered as another diagnostic criterion for diabetes. Only patients with autoimmune diseases or HIV were excluded from the study. Based on previous studies,^9^ and the 34% prevalence of DM in patients with TB for Iranians, we calculated the sample size minimum of 350 participants (*P* = 0.34, q = 0.66 and d = 0.051).

 For analyzing the data, descriptive indices such as mean, number and percentage were used. In order to study the factors associated with type II diabetes, chi-square test, t-test and logistic regression test were used. First, we performed the bivariate logistic regression for all variable and then we selected a variable when its related *P* value was less than 0.2. Afterwards, all variables selected in first step were included in the multivariate logistic regression. All analyses were done using the SPSS software version 11.5 and the significance level was set at *P* < 0.05.

## Results

 Among the 405 participants with a mean age of 51.14 ± 20.52 (range 19-92 years), 177 (43.7%) were males. In total, 236 (53.8%) were Iranian and 169 (41.7%) were Afghan; the mean age of the patients was 50.90 years (confidence interval 48.84, 52.96). The general prevalence of diabetes was 21.2%, of whom 3.5% were newly diagnosed. The patients’ demographic and clinical data are presented in Table 1. Accordingly, the mean age and body mass index was higher among patients with diabetes compared to those without it. Moreover, the prevalence of diabetes was higher among married patients and widowers compared to single participants. The prevalence of DM was lower among those with a higher educational status, dropping from 26.7% in illiterate cases to 11% in those with diploma and higher.

 Given the occupational status and DM, the highest prevalence of diabetes was seen among housewives followed by workers whereas the lowest prevalence was among businessmen. Also, 24.3% of cases with pulmonary TB had diabetes, whereas this figure was almost half (12.5%) in those with extrapulmonary TB.

**Table 1:** Comparison of Demographic and Clinical Variables in Two Groups with Diabetes and Without Diabetes

**Variables**		**Without Diabetes Mean±SD**	**With Diabetes Mean±SD**		***P*** ** Value**
FBS		92.88 ± 12.74	174.20 ± 55.90		< 0.001
HBA1C		5.73 ± 0.107	6.80 ± 0.26		< 0.001
Age		48.99 ± 21.69	58.13 ± 15.28		< 0.001
Body mass index		21.02 ± 8.40	23.59 ± 4.03		< 0.001
		**No. (%)**	**No. (%)**	**Total**	
Gender	Male	134 (81.2)	31 (18.8)	165 (43.6%)	0.320
	Female	164 (77.0)	49 (23.0)	213 (56.4%)	
Nationality	Iranian	175 (78.4(	48 (21.6(	223 (59%)	0.837
	Afghan	123 (79.3(	32 (20.7(	155 (41%)	
Marital status	Single	72 (93.5(	5 (6.5(	77 (20.4%)	< 0.001
	Married	170 (76.6(	52 (23.4)	222 (58.9%)	
	Widow/er	55 (70.5)	23 (29.5)	78 (20.7%)	
Educational level	Illiterate	113 (73.3)	41 (26.7)	154 (40.9%)	0.026
	Middle school	118 (79.2)	31 (20.8)	149 (39.6%)	
	≥ Diploma	65 (89.0)	8 (11.0)	73 (19.5%)	
Occupation	Housewife	113 (72.9)	42 (27.1)	155 (41%)	0.047
	Worker	51 (79.7)	13 (20.3)	64 (16.9%)	
	Businessman	134 (84.3)	25 (15.7)	159 (42.1%)	
Tobacco history	None	251 (78.9)	67 (21.1)	318 (85.7%)	0.714
	Cigar and Hookahs	43 (81.1)	10 (18.9)	53 (14.3%)	
TB type	Pulmonary	215 (75.7)	69 (24.3)	284 (76.3%)	0.019
	Extra-pulmonary	77 (87.5)	11 (12.5)	88 (23.7%)	
Treatment group	Group 1	270 (78.5)	74 (21.5)	344 (92.2%)	0.918
	Group 2	23 (79.3(	6 (20.7)	29 (7.8%)	
Treatment type	New case	271 (79.0)	72 (21.0)	343 (92.7%)	0.245
	Relapse	17 (70.8)	7 (29.2)	24 (6.5%)	
	Treatment after absenteeism	3 (100.0)	0 (0.0)	3 (0.8%)	

FBS, fasting blood sugar; TB, tuberculosis.

 The results of logistic regression for assessing the diabetes related factors in patients with TB, presented in Table 2, indicate that age, body mass index, nationality and TB type were associated with diabetes in patients with TB. In this study, patients with pulmonary TB were 3.32 times more likely to have diabetes compared to those with extrapulmonary TB.

**Table 2:** Results of Multivariate Logistic Regression Analyses in Those with and without Diabetes for Each Associated Variable

**Variables**		**B**	***P*** ** Value**	**OR**	**95% CI for (OR)**
Gender	Male	0.584	0.306	1.794	(0.587, 5.487)
Nationality	Iranian	0.765	0.021	2.149	(1.122, 4.117)
Occupation	Businessman	-	0.538	-	-
	Worker	0.555	0.275	1.742	(0.643, 4.721)
	Housewife	-0.080	0.864	0.923	(0.367, 2.318)
Disease type	Pulmonary	1.202	0.005	3.328	(1.44, 7.689)
Age	< 30	-	0.005	-	-
	30–45	-0.122	0.854	0.885	(0.241, 3.248)
	45–60	1.331	0.025	3.786	(1.183, 12.113)
	> 60	0.357	0.579	1.428	(0.406, 5.025)
Body mass index	< 18.5	-	0.000	-	-
	18.5–22.9	2.063	0.000	7.871	(2.888, 21.452)
	23–24.9	1.841	0.002	6.303	(1.956, 20.309)
	> 25	2.214	0.000	9.149	(3.182, 26.302)
Educational level	Diploma	-	0.295	—	
	Middle school	0.693	0.232	1.999	(0.641, 6.232)
	Illiterate	0.826	0.119	2.284	(0.81, 6.442)
Marital status	Widow/er	-	0.503	-	-
	Married	-0.823	0.241	0.439	(0.111, 1.738)
	Single	-0.254	0.529	0.776	(0.352, 1.709)

B, unstandardized coefficients; OR, odds ratio; CI, confidence interval for odds ratio.

 In order to insert the variables such as age and body mass index in the logistic regression model, we initially classified them as described in Table 2. It was observed that those between 45 to 60 years were 3.78 times more likely to have diabetes compared to those under 30 years of age. This difference was not significant for the other age groups.

 However, when comparing the body mass index, all subgroups showed a meaningful difference compared to age group under 18.5 years. The highest chance of having diabetes was seen in the > 25year age group with a 9.14-time higher risk.

## Discussion

 Based on the World Health Organization report, around 15 000 to 89 000 individuals are affected by TB each year.^7,12^ On the other hand, DM comorbidity with TB causes severe and irreversible complications. Most importantly, the presence of diabetes in patients with TB can prolong the treatment course along with increasing drug resistance.^13^ Therefore, diabetes screening in TB patients can result in early diagnosis and reduced complications.

 Based on our findings, the prevalence of diabetes in our study was 21.2%, which is higher than the prevalence in China (12.6%), Ethiopia (8.3%), Jammu, India (8.2%) and Uganda (8.5%).^4,11,14,15^ However, it is lower than the prevalence in Puducherry-India (29%), Tamil Nadu (25%), Taiwan (29.5%), southern Mexico (29.3%) and Kerela-India (44%).^10,16-19^

 Based on a study performed in Tehran, Iran in 2012, the prevalence of DM in TB patients was reported at 34.5% which is remarkably higher than our findings. This difference might be due to the fact that they omitted Afghan patients from their study, whereas the prevalence of DM in Iran is higher than Afghanistan. They also conducted their study in a research center in a hospital; 94% of their cases had pulmonary TB which may have caused a bias in patient selection and the overestimation of DM prevalence in TB cases.

 In the current study, we excluded human immunodeficiency virus cases as this disease affects both TB and DM whereas human immunodeficiency virus patients were included in the above-mentioned study.^9^ The reason for the difference in the DM prevalence in various countries may be related to the background differences in the population, the prevalence of DM in these nations and the quality and type of the screening methods used for DM diagnosis.

 These findings are indicative of the increased risk of diabetes in Iran. Therefore, changing the lifestyle and improving the health level can decrease the prevalence of DM. In addition, its prevalence in TB cases threatens the progresses made in controlling TB and evaluates the integrated health services approach for addressing the burden of both diseases. Among the DM cases in our study, 3.5% were newly-diagnosed cases whereas 17.7% were chronic cases; this figure was lower than the new cases reported from Puducherry-India (8.3%), Ethiopia (4.9%), Gujarat-India (4%) and Mexico (4.4%). However, it was higher than the newly-diagnosed cases reported from China (2.9%), Saluro-India (22%) and Kolar-India (2.9%).^4,11,19-23^ The difference in the DM diagnosed cases in this study might be related to disease prevalence, poor awareness and lack of access to health services. This further emphasizes the role of screening in TB cases for the early diagnosis and treatment of the two concurrent diseases.

 In general, various variables related to DM have been reported in TB patients such as age, marital status, educational level, type of TB, family history of DM, occupation and even in some cases, sex has been mentioned as an influencing factor.^3,22,24-26^

 In our study, marital status, educational level, occupation, age, body mass index and disease type were associated with DM in univariate analyses. However, in the multiple logistic regression analysis with the adjustment for other variables, only disease type, body mass index, age and nationality had a significant correlation.

 Our findings showed that pulmonary TB cases are 3.3 times more likely to have diabetes. However, the association between a smear-positive sputum and diabetes in TB patients is very common. In other studies, DM patients had the highest risk of smear-positive sputum which is indicative of the high risk of TB infection and its transmission in such patients. Also, one study reported that the presence of certain cavities in the chest x-ray was significantly associated with DM.^4,27,28^

 Body mass index was one of the other factors related to DM: an increase in body mass index was associated with an increased risk of DM. However, controversial results have been reported regarding the association between DM and body mass index in TB patients; some studies have reported no such correlation^13,19,28,29^ while other studies demonstrated a positive effect of body mass index on DM in such patients.^4^ The concurrent association of body mass index in DM and TB is very complicated. While an increase in body mass index increases the risk of DM, yet overweight and obesity act as a protective factor for TB. Weight loss with the aim of reducing the risk of DM results in the lack of protective metabolic control against TB. On the other hand, the presence of TB increases the risk of hyperglycemia but causes weight loss. Nevertheless, these equations may vary in different individuals.^28^ Therefore, it may be best to have normal weight if both diseases are present; as mentioned in this study, the chance of infection in those with normal weight (body mass index: 23–25) is lower than the obese or low weight individuals.

 In almost all studies, similar to ours, age has been an influential factor for diabetes in those affected by TB.^4,10,28^ With aging, the body’s immune system declines; therefore, aging is a risk factor for both DM and TB. However, elderly people are usually less literate and so their awareness regarding such diseases, their association and mechanisms is poorer, which can affect the increased risk of infection in such cases.

 In our studied TB cases, Iranians had a 2.14-time higher risk of DM compared to Afghans. In addition to the underlying genetic issues in the two nationalities that may be effective,^30^ based on the International Diabetes Federation report, the prevalence of DM in general in Afghanistan is lower compared to Iran.^31^ Moreover, one can also point out that most Afghans live in the suburbs and do not have a good socio-economic status and therefore are obliged to work harder than Iranians. On the other hand, a high socio-economic status and low activity levels are referred to as diabetes-related factors in several studies. Taken together, these factors can lower the risk of DM in Afghans in comparison to Iranians.^24,25,32^

 Regarding the main limitations of this study, considering that DM prevalence in TB patients in urban areas may differ from rural areas, our study only included the urban population. In addition, the undesirable societal view of the use of cigarettes, alcohol and pan consumption habits may have affected the non-response in such patients along with the research outcomes regarding the behavioral risk factors of DM and TB.

 In the current study, hemoglobin A1c and fasting blood glucose testing were used for screening. However, among glucose tolerance test, hemoglobin A1c, Fasting blood glucose and random blood glucose, no absolute preferential method has yet been introduced for screening DM in TB patients. Even the best screening time is not evident yet. For example, screening at the beginning of TB diagnosis can be misleading as TB, as an infectious disease, can temporarily increase the blood glucose level and an infection-related hyperglycemia can be misdiagnosed as diabetes.^3^ On the other hand, primary screening for DM has certain privileges such as starting DM treatment, patient’s education and hyperglycemia correction which can potentially affect the TB treatment outcome. Some studies suggest that TB patients should be observed for the blood glucose level throughout the treatment course. However, in other studies, DM screening at the time of TB diagnosis and 3 months after treatment initiation has been considered as adequate.^26,27^ Nevertheless, fundamental, practical and operational research is recommended to yield standard management guidelines for achieving the best timing and screening methods for predicting, preparing and increasing the chance of reducing the dual burden of DM and TB. The Collaborative Framework for the Care and Control of Tuberculosis and Diabetes proposes bidirectional screening and joint management. Screening patients with TB for DM also yielded a high prevalence of DM ranging from 1.9% to 35%.

 The concurrence of both diseases represents a risk for the possible spread of TB worldwide as well as serious implications for TB control and the achievement of the Millennium Development Goals. In conclusion, this study revealed that prevalence of DM was 21.2% in patients with TB. In multivariate logistic regression analyses, TB type, body mass index, age and nationality had a significant association with DM.

 Health education about DM, TB and their comorbidity can reduce both of them. Finally, it must be borne in mind that higher prevalence does not necessarily mean higher risk as it could be because of the higher survival of patients. On the other hand, one of the limitations in our study was that we could not verify the real cause and effect relationship between TB and diabetes.
